# Dual repression of endocytic players by ESCC microRNAs and the Polycomb complex regulates mouse embryonic stem cell pluripotency

**DOI:** 10.1038/s41598-017-17828-7

**Published:** 2017-12-14

**Authors:** Ridim Dadasaheb Mote, Gaurang Mahajan, Anup Padmanabhan, Ramaraju Ambati, Deepa Subramanyam

**Affiliations:** 1 National Centre for Cell Science, SP Pune University, Ganeshkhind, Pune, 411007 India; 2 Mechanobiology Institute, National University of Singapore, 5A Engineering Drive 1, Singapore, 117411 Singapore

## Abstract

Cell fate determination in the early mammalian embryo is regulated by multiple mechanisms. Recently, genes involved in vesicular trafficking have been shown to play an important role in cell fate choice, although the regulation of their expression remains poorly understood. Here we demonstrate for the first time that multiple endocytosis associated genes (EAGs) are repressed through a novel, dual mechanism in mouse embryonic stem cells (mESCs). This involves the action of the Polycomb Repressive Complex, PRC2, as well as post-transcriptional regulation by the ESC-specific cell cycle-regulating (ESCC) family of microRNAs. This repression is relieved upon differentiation. Forced expression of EAGs in mESCs results in a decrease in pluripotency, highlighting the importance of dual repression in cell fate regulation. We propose that endocytosis is critical for cell fate choice, and dual repression may function to tightly regulate levels of endocytic genes.

## Introduction

Mammalian development requires exquisitely controlled molecular mechanisms to ensure accurate fate choice in a spatial and temporal manner. Such fate choices have been shown to be regulated by a variety of factors including cellular trafficking and endocytosis^1^, with disruption of the endocytic network resulting in an impairment of early vertebrate development^1–4^. The maintenance of pluripotency of human ESCs and normal development of zebrafish have been shown to be dependent on the accurate endocytic trafficking of E-cadherin^5,6^. The endosomal protein, Asrij, has been shown to regulate mESC pluripotency, by acting as a scaffold for STAT3 activation^7^. Further, during the process of somatic cell reprogramming, alterations in endocytic gene expression have been observed, with certain endocytic pathways functioning as barriers in cell fate conversion^8,9^. Despite these lines of evidence, the regulation of endocytic gene expression in stem cells (by transcriptional and/or post-transcriptional mechanisms), and the precise role of endocytosis in the regulation of pluripotency remains unknown.

In addition to the recent findings demonstrating the regulation of ESC pluripotency by endocytic mechanisms, it is well appreciated that a delicate balance between the action of the core pluripotency transcriptional circuit, along with the poised yet repressed state of key developmental regulators by the Polycomb repressive complex (PRC) is important^10–13^. Further, it is also well known that specific, non-coding RNAs, particularly microRNAs belonging to the ESCC family, are also expressed and play a key role in regulating various cellular functions in ESCs^14,15^.

Here, we identify for the first time a dual-repressive molecular circuit involving the Polycomb Repressive Complex (PRC) and ESCC microRNAs that regulate the expression of endocytosis-associated genes (EAGs) in mESCs. We have identified a large number of EAGs that are bound and repressed by the PRC in mESCs. Interestingly, a subset of these genes are further subjected to post-transcriptional regulation by miR-294, a member of the ESCC family of miRNAs, indicating the existence of a ‘dual mechanism’ of gene repression. Knockdown of *Ezh2* or *Suz12* results in a collective derepression of these genes in mESCs, while ectopic expression of miR-294 results in a reduction of their expression. We further demonstrate using two of the EAGs, namely *Cav1* and *Cdh2*, that their ectopic expression results in decreased pluripotency of mESCs, indicating a critical requirement for the repression of specific endocytic pathways for cell fate maintenance. We also demonstrate evolutionary conservation of EAGs in early development, using *C*. *elegans* embryos. We show that the knockdown of *Cav1* in these embryos results in a delay in development. We propose that the existence of a dual repressive mechanism emphasizes the requirement for a failsafe regulatory mechanism during development.

## Results

### Identification of transcriptional regulation of endocytic networks in mESCs

An intricate transcription factor network functions to regulate the pluripotency of ESCs^10,16^. To identify whether the pluripotency network regulates the expression of specific endocytic pathways, or endocytosis-associated genes in mESCs, we integrated two previously published datasets; namely i) ChIP-seq data on genome-wide transcriptional regulation in mESCs by an ESC-specific regulatory network comprising 13 transcription factors (TFs), and the epigenetic regulator, SUZ12 from Chen *et al*.^16^; and ii) expression profiling data from mESCs and mouse embryonic fibroblasts (MEFs)^17^.

**Figure 1:**
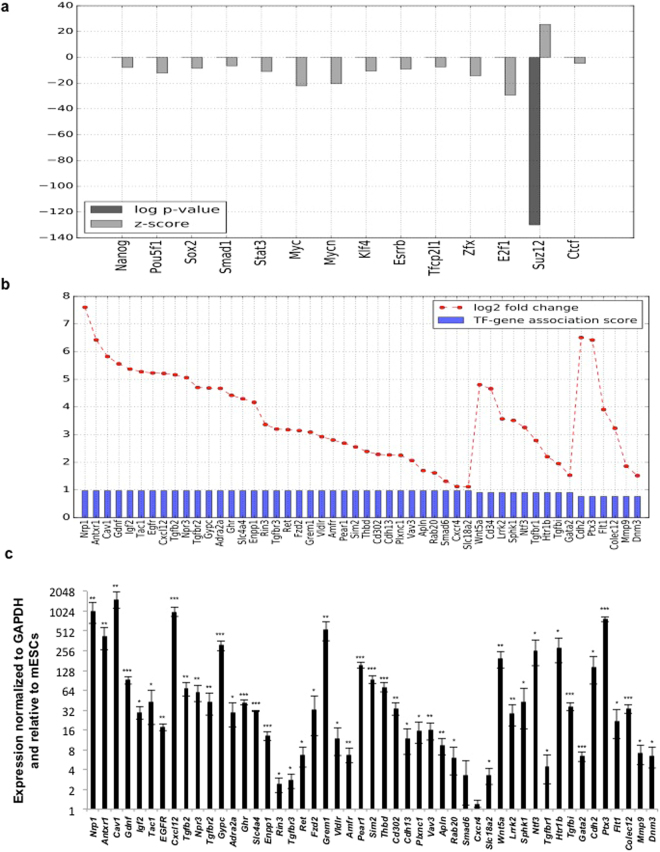
*In silico* identification of transcription factors and transcriptional regulators that regulate endocytic gene expression in pluripotent and differentiated cells. (**a**) Bar chart displaying the statistical association between gene sets bound by different transcriptional regulators (from ChIP-seq binding data in Chen *et al*.), and the set of genes whose expression levels are 2-fold higher in MEFs relative to mESCs. Differentially upregulated genes are significantly over-represented in the gene targets of SUZ12 (indicated by a low p-value and high Z-score). (**b**) Bar chart showing the 50 genes with known association with endocytosis, whose promoter regions are bound by SUZ12 and are upregulated in MEFs relative to mESCs. The interaction score between SUZ12 and each gene, as well as the log2 fold expression change of each gene, are shown. (**c**) RT-qPCR analysis of SUZ12 bound endocytic genes in mESCs and MEFs. mRNA expression is normalized to *Gapdh*, and further normalized to expression in mESCs. Error bars represent mean ± S.D for experiments in triplicates (N = 3). *p < 0.05; **p < 0.01; ***p < 0.001 by Students T-test.

Genes that were expressed in MEFs and repressed in mESCs by >2-fold (at FDR < 0.001), were analyzed for enrichment of any of the 13 TFs or SUZ12. Our analysis highlights an important role for SUZ12 in global transcriptional repression in the pluripotent state, which is relieved in the differentiated state (Fig. 1a). This is represented in the bar chart in Fig. 1a, which shows that the target set of SUZ12 (i.e. the subset of genes whose upstream promoter regions were shown to be bound by SUZ12 in the ChIP-seq data from Chen *et al*.)^16^, is significantly enriched for genes whose expression levels are >2-fold in MEFs relative to mESCs. Consistent with this, we find that SUZ12 expression is downregulated in MEFs relative to mESCs (Supp. Figure 1b,c). Of the SUZ12-bound genes which are >2-fold higher in MEFs compared to mESCs, there are 50 genes which are known to have putative roles in endocytosis or are associated with this cellular process, henceforth referred to as endocytosis associated genes (EAGs) (this list was manually put together by combining lists detailing genes associated with endocytosis from KEGG and GO). These are separately displayed along with their expression change values in Fig. 1b. To further validate these observations, the expression of EAGs was determined using RT-qPCR in mESCs and MEFs (Fig. 1c). Consistent with data shown in Fig. 1b, EAG expression in MEFs was higher compared to mESCs (Fig. 1c). Thus, our analysis points to a possible role for SUZ12 in actively repressing endocytosis-related processes during the maintenance of pluripotency through histone methylation. To further validate our approach, we checked to see if genes that are expressed >2-fold in mESCs relative to MEFs were enriched for TFs that are known members of the pluripotency network. We find that 11 of the 13 sequence specific TFs in the ES cell-specific regulatory network are significantly associated with this gene set (with highly significant over-representation p-values as well as z-scores) (Supp. Figure 1a). This supports and validates the known role of these TFs in the maintenance of the pluripotent state, and also suggests that their role as transcriptional activators in global gene regulation is diminished following differentiation.

### Repression of endocytosis-associated genes by the PRC2 complex

**Figure 2:**
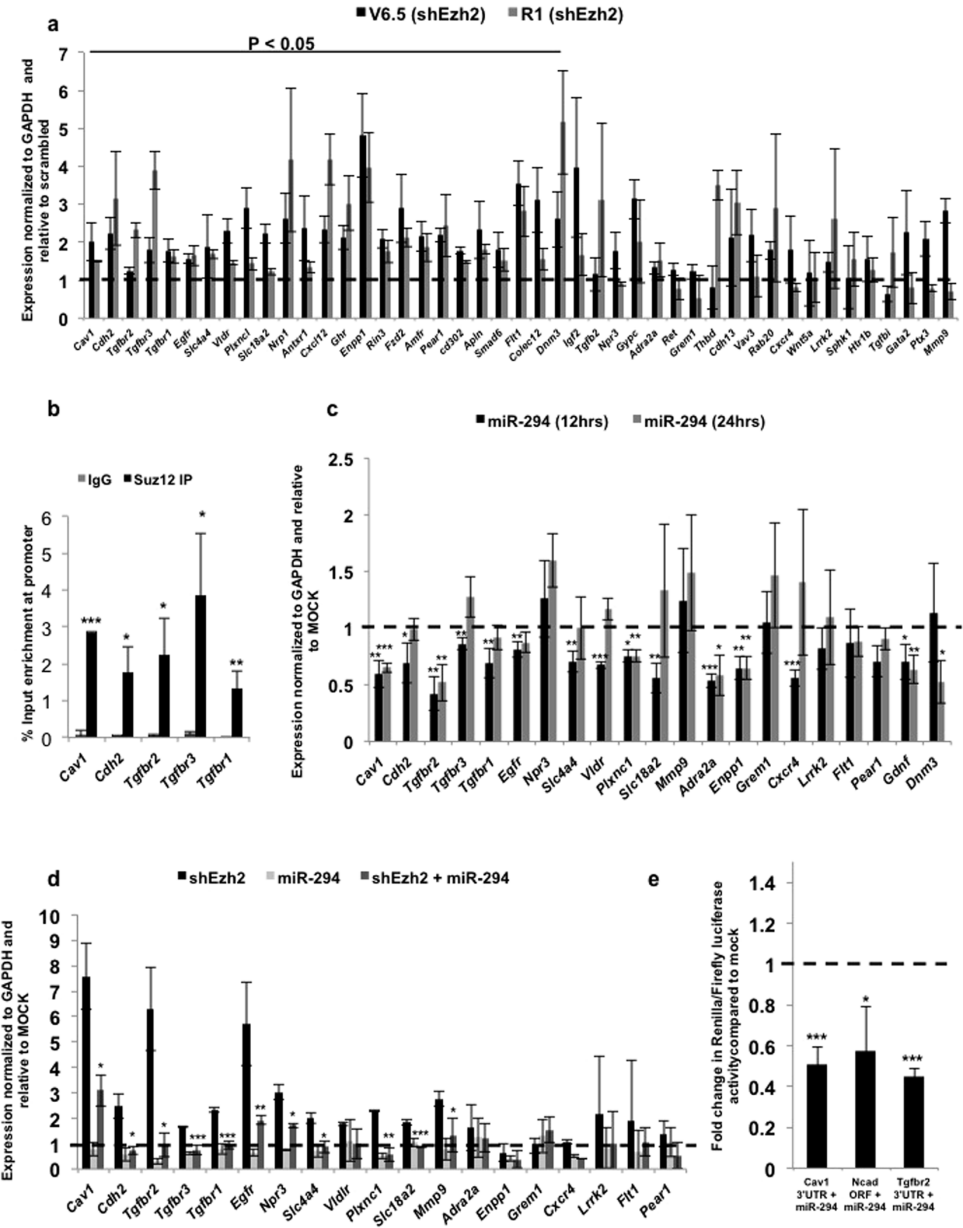
Dual regulation of endocytic genes by the PRC2 complex and ESCC miRNAs. (**a**) RT-qPCR analysis of PRC2 target genes upon *Ezh2* knockdown in V6.5 and R1 mESCs. mRNA expression is normalized to *Gapdh* and represented relative to scrambled shRNA. Scrambled shRNA is represented as a dashed line at 1. (**b)** Graph showing enrichment of SUZ12 at *Cav1*, *Cdh2*, *Tgfbr1*, *Tgfbr2* and *Tgfbr3* promoters by chromatin immunoprecipitation using IgG or SUZ12 specific antibody. (**c)** RT-qPCR analysis for endocytic gene expression in MEFs in the presence of exogenous miR-294, 12 and 24 hrs post-transfection. Mock is represented as a dashed line at 1. (**d)** RT-qPCR analysis of PRC2 target genes in *Dgcr8* KO mESCs upon *Ezh2* knockdown, and in the presence of exogenous miR-294. mRNA expression is normalized to *Gapdh* and represented relative to scrambled shRNA. Scrambled shRNA is represented as a dashed line at 1. (**e)** Luciferase analysis of *Cav1* 3′UTR, *Cdh2 (N-Cad)* ORF and human *Tgfbr2* 3′UTR. MEFs were co-transfected in the presence or absence of miR-294 and respective pSICHECK2 vectors. Renilla luciferase activity levels were normalized to firefly luciferase, which served as an internal control. Mock transfection level is represented as a dashed line at 1. For all experiments, error bars represent mean ± S.D for experiments in triplicates (N = 3). *p < 0.05; **p < 0.01; ***p < 0.001 by Students T-test.

SUZ12 is a component of the PRC2 complex, which has four subunits: SUZ12 (zinc finger containing protein), EED, EZH1 or EZH2 (SET domain containing protein with histone methyl transferase activity) and RBAP48 (histone binding domain containing protein). The PRC2 complex has histone methyltransferase activity and trimethylates histone H3 on lysine 27 (i.e. H3K27me3), a mark of transcriptionally silent chromatin^18^. Knockout of *Suz12*, *Ezh2*, *Eed* results in embryonic lethality at E7.5–8.5 with major defects in gastrulation, with mESCs failing to properly differentiate^19^. In order to determine whether the PRC2 complex was indeed involved in regulating expression of EAGs, (shown in Fig. 1b,c), the catalytic component of the PRC2 complex, *Ezh2*, was knocked down in two mESC lines, V6.5 and R1, using lentiviral shRNA constructs (Supp. Figure 2a,b). The expression of 45 EAGs was determined using RT-qPCR following *Ezh2* knockdown in V6.5 and R1 mESCs (Fig. 2a). Expression of 25 out of these 45 genes was significantly and consistently derepressed upon *Ezh2* knockdown (Fig. 2a). Similar results were also obtained upon the knockdown of *Suz12*, a zinc finger-containing DNA binding component of the PRC2 complex (Supp. Figure 2c,d). We further validated that the regulation of a handful of these EAGs was due to a direct association of the PRC2 complex at their promoter and not due to an indirect effect by performing chromatin immunoprecipitation (ChIP) for a few EAGs. SUZ12 showed strong binding to the *Cav1*, *Cdh2*, *Tgfbr1*, *Tgfbr2* and *Tgfbr3* promoters as determined by ChIP followed by RT-qPCR (Fig. 2b). Together, these results indicate that the expression of a number of EAGs is indeed repressed in mESCs through the direct action of the PRC2 complex.

### Dual regulation of EAG expression by the ESCC miRNA family and the PRC2 complex

The ESCC family of microRNAs (miRNAs) are a family of conserved miRNAs that are highly expressed in ESCs, play a role in cell cycle regulation, and enhance the efficiency of somatic cell reprogramming^8,15,20^. miRNAs are small non-coding RNAs that post-transcriptionally regulate gene expression through complementary binding of their seed sequence with a seed match present in the target mRNA^21^. Intriguingly, of the 50 endocytic genes whose promoter regions are bound by Suz12 (Fig. 1b), we found that 21 genes also had seed matches for miR-294, a member of the ESCC family of miRNAs, either in their 3′UTR or ORF (Supp. Table 1). To validate if these seed matches were indeed functional targets of the ESCC miRNA family, the ESCC miRNA, miR-294 was exogenously introduced into MEFs, cells that are naturally devoid of these miRNAs. 16 out of 21 EAGs were found to be functional targets of miR-294 as their expression significantly decreased upon overexpression of miR-294 (Fig. 2c), indicative of a role of these miRNAs in regulating the stability of EAG transcripts. To further verify this regulation, we utilized the previously published *Dgcr8* KO ESC line, which lacks all mature miRNAs^22^. Introduction of synthetic miR-294 into *Dgcr8* KO mESCs resulted in the decreased expression of 11 out of 21 EAGs (Fig. 2d). Knockdown of *Ezh2* (Supp. Figure 2e) resulted in an increase in expression, while a combination of *Ezh2* knockdown along with exogenous supply of miR-294 resulted in intermediate levels (Fig. 2d). This powerfully demonstrates the responsiveness of specific EAGs to dual regulation by the PRC2 complex as well as by miR-294.

To demonstrate binding and regulation of EAGs by the ESCC family of miRNAs, the 3′UTRs of *Cav1*, *Cdh2* and human *Tgfbr2* (which shows a high degree of conservation with the mouse *Tgfbr2* 3′UTR) were amplified and cloned downstream of renilla luciferase in the pSiCHECK2 vector. The *Cav1* 3′UTR contains one seed match (6-mer), *Cdh2* ORF contains one seed match (7-mer) (Supp. Table 1), and the *Tgfbr2* 3′UTR contains three (7-mer) and two (6-mer) seed matches for the ESCC miRNAs. A significant downregulation of renilla luciferase activity in the miR-294 transfected samples was observed with all three constructs, further validating their regulation by the ESCC miRNAs (Fig. 2e). Introduction of miR-294 into HEK293 cells also resulted in a decrease of CAV1 and CDH2 protein levels (Supp. Figure 3a,b). Together these experiments validate a role for the ESCC microRNAs in regulating the stability of a number of EAGs in mESCs.

### Exogenous expression of EAGs results in a decrease in mESC pluripotency

To interrogate whether the repression of EAGs was essential for the maintenance of pluripotency, we chose two candidates, namely *Cav1* and *Cdh2* out of the 12 dual regulated genes. CAV1 is an integral member of the caveolae-mediated endocytic pathway, and has been shown to regulate specific signalling pathways that initiate at the membrane, including WNT and TGFβ^23–25^. CAV1 works in conjunction with its partner, CAVIN1 for normal caveolar biogenesis^26^. CDH2 is a single-pass transmembrane protein, whose compartmentalization into different endocytic compartments has been shown to play an important role during mouse cerebral cortex development^27^.

**Figure 3:**
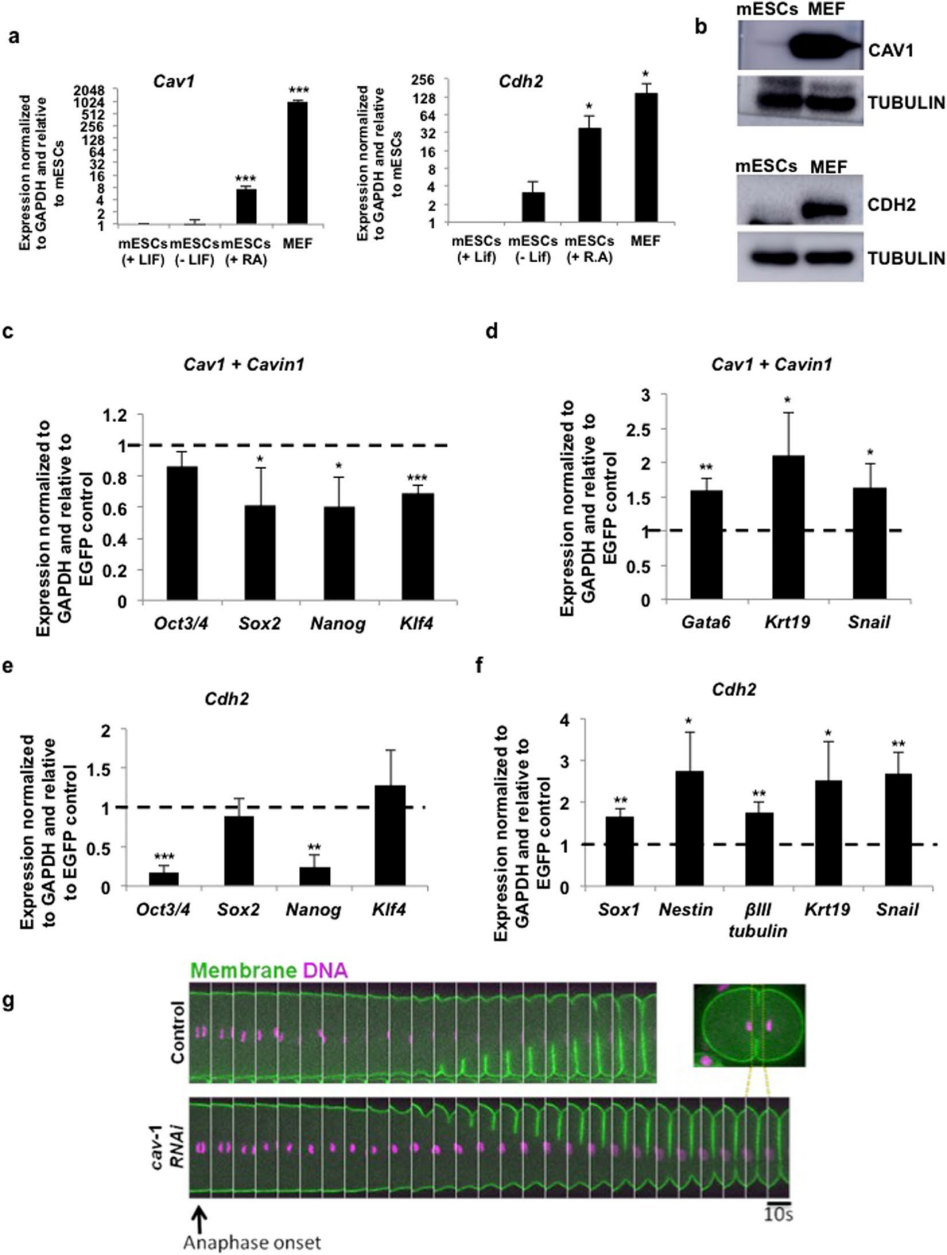
Misexpression of *Cav1/Cdh2* in mESCs provide a differentiation cue in mESCs. (**a)** RT-qPCR analysis for *Cav1* and *Cdh2* mRNA expression in the following samples: mESCs cultured in the presence of LIF, mESCs cultured in the absence of LIF for 72hrs, mESCs cultured with retinoic acid for 96hrs, and in MEFs (N = 3). (**b)** Western blot showing expression of CAV1 and CDH2 in mESCs and MEFs (N = 3). (**c–f)** RT-qPCR analysis of pluripotency markers (**c**,**e**) and differentiation markers (**d**,**f**) in mESCs upon overexpression of *Cav1*, along with *Cavin1* or *Cdh2*. For all experiments, error bars represent mean ± S.D for experiments in triplicates (N = 3). *p < 0.05; **p < 0.01; ***p < 0.001 by Students T-test. (**g)** Time-lapse confocal images of the equatorial plane showing ingression of the first cytokinetic furrow in control and *cav-1(RNAi) C*. *elegans* embryos co-expressing GFP::PLC1δ –PH (membrane marker) and Histone::mCherry (DNA marker). The furrow takes a longer time to complete ingression upon CAV-1 depletion. Inset shows the region of interest (ROI) made into a montage.

Caveolin-mediated endocytosis is an important route for the internalization of proteins and molecules within a cell^23^. Further, in mammary stem cells, the loss of *Cav1* has been shown to result in an increase in the stem cell population^25^. The other dual regulated gene, *Cdh2*, enhances mESC differentiation upon supplementation with FGF2^28^. Moreover, CDH2-based substrates have been used for mESC and iPSC differentiation towards the neural lineage^29,30^. The dual repression of *Cav1* and *Cdh2* in mESCs suggests that their repression may be essential to the maintenance of the pluripotent state. To test this, we checked the expression of *Cav1* and *Cdh2*, and found their expression to be very low at both the mRNA and protein levels in mESCs, with expression increasing upon differentiation (Fig. 3a,b). Similar to *Cav1*, *Cavin1* that acts along with *Cav1* for caveolin-mediated endocytosis, had lower expression in mESCs, with levels increasing upon differentiation (Supp. Figure 3c). Immunostaining of mESCs using antibodies specific to CAV1 and CDH2 showed very low expression, whereas MEFs showed expression of CAV1 and CDH2 (Supp. Figure 3d,e).

In order to demonstrate that the dual repression of *Cav1* and *Cdh2* is critical for the maintenance of pluripotency, we ectopically expressed *Cav1* along with *Cavin1* (Supp. Figure 3f,g), or *Cdh2* (Supp. Figure 3h), in mESCs. Overexpression of *Cav1* along with *Cavin1*, or *Cdh2* caused a significant decrease in the expression of the pluripotency markers (Fig. 3c,e), along with a significant increase in the expression of differentiation markers (Fig. 3d,f). Together, our results demonstrate that the regulation of endocytosis associated genes is important for the maintenance of pluripotency.

Caveolin has been implicated in regulating the meiotic progression during oocyte development in *C*. *elegans* ^31^. CAV-1 is expressed in the germline and early embryos in *C*. *elegans* ^31^. We therefore investigated the role of CAV-1 in the development of *C*. *elegans*, post fertilization. To this end we carried out live imaging of control and *cav-1 RNAi* embryos co-expressing GFP fused to PLC1**δ**-PH and mCherry fused to Histone to mark membrane and DNA respectively. We monitored the first cell division in the embryonic 1-cell stage. While initiation of furrow was similar in control and *cav-1 RNAi* embryos, ingression of the cytokinetic furrow was markedly delayed in *cav-1 RNAi* embryos compared to the control (Fig. 3g), indicative of a developmental delay as early as the 1–2 cell stage. Together, these results demonstrate the evolutionary conservation of the importance of EAGs in early embryonic development. The existence of a dual repressive mechanism, where one type of repression may function as a back-up in case the other fails, further strengthens the notion that accurate control of endocytic pathways plays a key role in cell fate maintenance.

## Discussion

The involvement of cellular trafficking in the regulation of the pluripotent state is a relatively new concept. While alterations in the expression of endocytic genes during cell fate switching have been observed during the process of reprogramming^8^, the exact mechanism of regulation of these genes remains unknown. Here we show for the first time that specific endocytic genes are repressed in mESCs compared to differentiated cells (Fig. 1b,c). This repression occurs at two levels— at the transcriptional level through the action of the repressive methyltransferase, PRC2, and at the posttranscriptional level by the ESCC family of miRNAs (Fig. 2). Together, these two mechanisms ensure that the expression of these genes remains repressed in the pluripotent state. While there are isolated examples in the literature describing similar mechanisms^32,33^, this is the first time that such a dual regulation has been described in ESCs with respect to cellular trafficking and the regulation of the pluripotent state.

While *Cav1* is a major endocytic player that is dually repressed, a number of other molecules that are associated with endocytosis are similarly repressed. These include *Cdh2* and members of the TGFβ pathway, including *Tgfbr1*, *Tgfbr2* and *Tgfbr3* (Figs 2, 3). All these EAGs have been previously shown to play an integral part during differentiation. The caveolin-mediated endocytic pathway, regulates the activity of signalling pathways such as WNT and TGFβ, both of which are known to play a central role in ESC pluripotency^23–25^. Endocytosis of CDH2 in an AP-2 adaptor complex dependent manner has been shown to be essential for neurite outgrowth and circuit formation^34^. Inhibition of RAB5 (early endosomal marker), or RAB11 (recycling endosome marker), showed defects in CDH2 trafficking, which resulted in neuronal migration defects during mouse cerebral cortex development^27^. The TGFβ pathway has been shown to affect the pluripotency of stem cells^8,35,36^. The signalling outcome of the TGFβ pathway is also tightly regulated in an endocytic pathway-dependent manner. Clathrin-mediated endocytosis promotes TGFβ induced SMAD activation, while lipid rafts/caveolae facilitate the degradation of TGFβ receptors and therefore turn off TGFβ signalling^24,37^.

Interestingly, overexpression of two candidate EAGs, *Cav1* and *Cdh2* result in a decrease in pluripotency and an upregulation of differentiation markers (Fig. 3). Knockdown of *cav-1* in *C*. *elegans* also results in a delay in cytokinetic furrow progression (Fig. 3). While these genes singly do not cause a complete shift in cell fate, they do cause a decrease in pluripotency upon overexpression, suggestive that perhaps collectively these genes, along with others that are similarly regulated, may be capable of facilitating more drastic transitions in cell fate.

Apart from the dual regulation that we describe in this study, it is possible that additional modes of regulation may operate to control the levels of expression of these genes during development. Indeed previous reports indicate that CAV1 itself can also undergo ubiquitination, followed by lysosomal degradation^38^. It is thus likely that other EAGs may also be subject to additional mechanisms of regulation during development.

**Figure 4:**
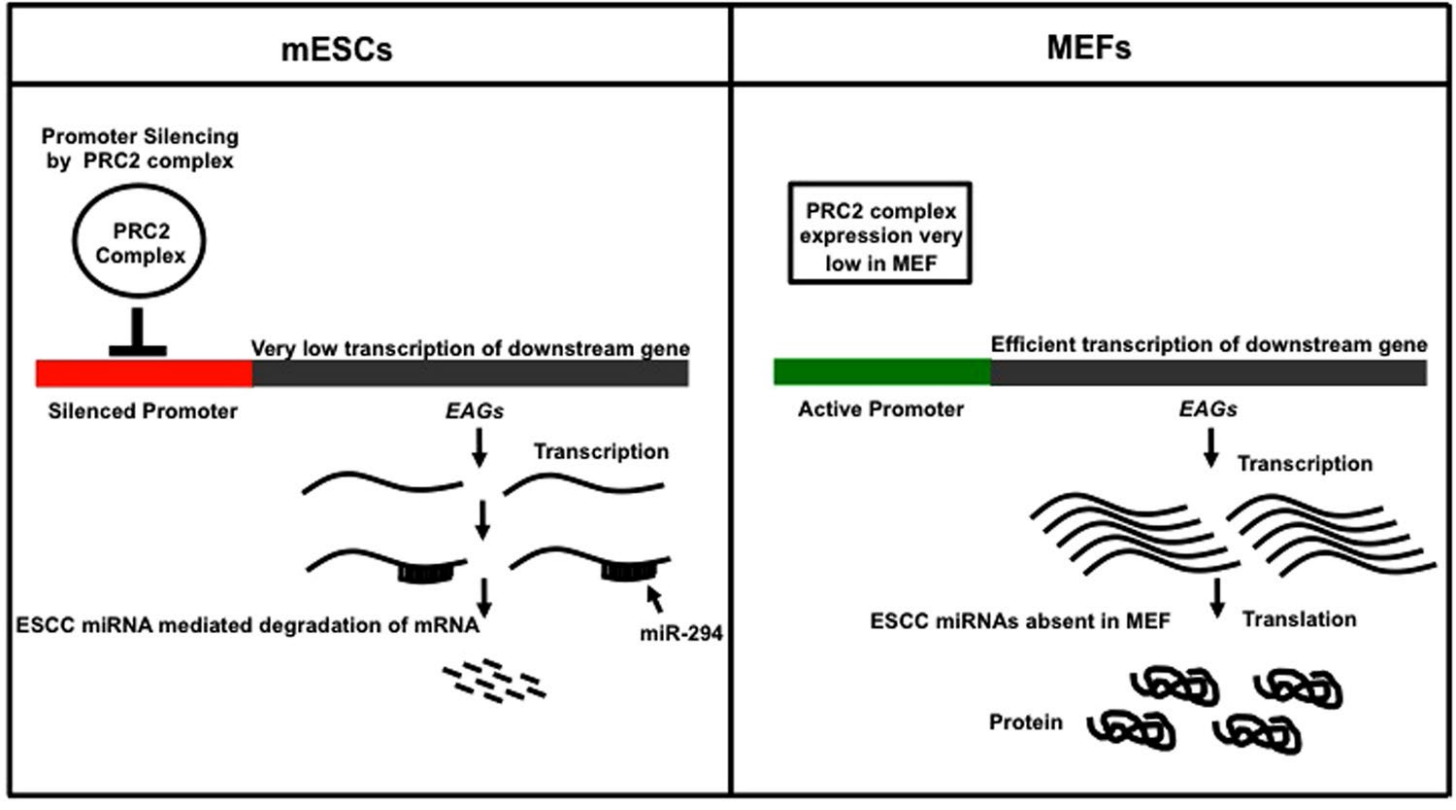
Dual repression of endocytic players in pluripotent mESCs. Model showing dual repression of endocytic players by SUZ12 (transcriptional repression at the promoter), and ESCC microRNAs (post-transcriptional regulation) in mESCs, resulting in a decreased expression of these genes. In differentiated cells, both these modes of repression are relieved, resulting in expression of these genes.

In conclusion, our data stresses the importance of regulation of specific endocytic pathways in cell fate maintenance. We propose a model wherein key genes (such as those that are involved in endocytosis) that can drive cell fate changes, are kept under a tight check by both transcriptional and post-transcriptional mechanisms (Fig. 4). We speculate that dual repression functions as a back-up mechanism to prevent leaky expression of genes that may play a role in facilitating a shift between cell fates.

## Materials and Methods

### Mouse embryonic stem cell culture

V6.5 or R1 mouse embryonic stem cells were cultured on tissue culture grade plastic plates coated with 0.2% gelatin. They were maintained in Knockout DMEM supplemented with 15% heat-inactivated fetal bovine serum, 0.1 mM beta mercaptoethanol, 2mM L-glutamine, 0.1 mM nonessential amino acids, 5000 U/ml penicillin/streptomycin, and 1000 U/ml LIF (ESC medium). Cells were passaged every 3 days using trypsin.

### Mouse embryonic fibroblast culture

Mouse embryonic fibroblasts were isolated from mouse embryos at e13.5 dpc from CF1 strain of mice. Mouse embryonic fibroblasts were maintained in DMEM supplemented with 10% heat inactivated fetal bovine serum, 0.1 mM beta mercaptoethanol (GIBCO-BRL), 2mM L-glutamine, 0.1 mM nonessential amino acids, 5000 U/ml penicillin/streptomycin (MEF medium). Cells were passaged every 4–5 days using trypsin.

### SiRNA preparation

Suz12 was amplified from V6.5 mESCs cDNA using Suz12 specific primers having flanking T7 sequence. T7 primer was then used to amplify the entire sequence. T7 RNA polymerase was used to perform *in-vitro* transcription, and the double stranded RNA generated was digested using RNAse III^39^. Non targeting siRNA was prepared using GFP as a template.

### Transfection

6 × 10^3^ cells were plated on gelatin-coated 24 well tissue culture grade plastic plates. The following day, media was changed and cells were transfected using 50 nM of respective siRNA or miRNA using DharmaFECT1 (Thermo Scientific Catalog no. # T-2001-03) in the appropriate medium. Media was changed every 24 hours. Transfection of plasmids was carried out using Lipofectamine 2000 (Invitrogen), as per manufacturer’s instructions. pEGFP-PTRF (ADDGENE NO. 68401) and pEGFPN1-Ncad-GFP (ADDGENE NO. 18870) were obtained from Addgene. pEGFPN1-Cav1 was a kind gift from Dr. Nagaraj Balasubramanian. For *Cdh2* overexpression, mESCs were grown in E.S media without LIF supplemented with Retinoic Acid (10^−8^M) and 5 μg/ml FGF2.

### Immunocytochemistry and imaging

3 × 10^4^mESCs or 2 × 10^4^ MEFs were plated on gelatin coated coverslips in a 24 well plate. The following day, cells were washed with PBS, fixed with 4% paraformaldehyde at room temperature for 20 min, and permeabilized with 0.1% Triton X-100 in PBS for 5 min. After blocking in 5% FBS and 5% bovine serum albumin in PBS for 1 h, cells were incubated with primary antibodies appropriately diluted in blocking buffer overnight at 4 °C. The following day, cells were washed with PBS for 30 min at room temperature and incubated with fluorescently conjugated secondary antibodies for 1 h. Post washing, nuclei were stained with DAPI. After 2 washes, coverslips were mounted onto glass slides and analyzed using a Zeiss 510 laser-scanning confocal microscope.

### RNA isolation and Real time PCR

Total RNA was isolated from mESCs or MEFs using TRIzol as per manufacturer’s instructions. Complementary DNAs (cDNA) were synthesized using Superscript-III first-strand synthesis system for RT-PCR as per manufacturer’s instructions. Gene-specific primers for RT-qPCR were designed using ABI Primer Express 3.0 software (sequences provided in Supp. Table 2). The quantitative RT–PCR reactions were done using ABI power SYBR Green PCR master mix and reactions were run on the ABI qPCR system, 7900 HT.

### Western blotting

Total proteins were extracted from cells using RIPA buffer containing proteinase inhibitors on ice followed by centrifugation at 12,000 rpm for 20 min at 4 °C. Protein concentration was measured using Bradford’s reagent. Equal quantity of total protein were subjected to SDS-PAGE under reducing conditions followed by transfer to PVDF membrane. After transfer, the membrane was blocked using 5% BSA in TBS. Post blocking, the membrane was incubated at 4 °C overnight with the appropriate primary antibody. After 3 × 10 min wash in Tris-buffered saline (1X TBS) containing 0.1% Tween-20 (TBS-T), the membranes were incubated with an HRP-conjugated secondary antibody (1:1000) for 1 hour at room temperature. NovexECL reagent was added to the membranes and images were captured post exposure using a chemi-doc system (GE Healthcare, Catalog no. AI600). Western blot intensity was documented using the ImageJ software.

### Luciferase Reporter Assay

*Cav1* 3′ UTR and *Cdh2 (N-Cad)* ORF were amplified from MEF cDNA and cloned into the NotI and XhoI sites in psiCheck™-2 vector (Promega). For transfection, 6000 MEFs were plated per well in a 96 well plate. The following day, MEFs were transfected with 100 nM of mmu-miR-294-3p (Ambion, The RNA company, Cat# 4464066 ID: MC10865) using Dharmafect1 (Dharmacon, Thermo Fisher Scientific). After 3 hours, media was changed and MEFs were transfected with either 200ng of psiCheck-2 Cav1 3′ UTR vector or psiCheck-2 Cdh2 ORF or psiCheck-2-Tgfbr2-wt3′UTR (ADDGENE NO. 31882). Luciferase assay was performed after 24 hrs of transfection using Dual-Glo® Luciferase Assay System (Promega, REF: E2920) on a GloMax®-Multi Detection System (Promega). Ratio of Renilla luciferase activity to firefly luciferase was calculated for each experiment.

### Regulatory TF data

Data for genome-wide transcriptional regulation in ES cells was independently obtained from Chen *et al*.^16^. This regulatory network was assembled by pooling together the genome-wide ChIP-seq profiles (binding sites) for 13 sequence-specific transcription factors (TFs) and 2 transcriptional regulators (P300 and SUZ12). Each pair of gene and TF in the network has also been assigned an association score between 0 and 1, reflecting the relative proximity of the TF binding site to the TSS.

### TF enrichment analysis

In order to identify putative transcription factors whose differential activity can account for the gene expression differences between ES and MEF cells, we integrated information about the differentially expressed genes (DEGs) with the ES cell-specific transcriptional regulatory network. DEGs were identified using previously published microarray expression profiles for mouse embryonic stem cells (mESCs) and mouse embryonic fibroblasts (MEFs)^17^. The expression profiles along with the associated annotation data, were downloaded from the GEO database (GEO accession number: GSE8024). This dataset consists of three replicates of WT ES cells and two of MEFs, profiled with the Affymetrix Mouse Genome 430 2.0 Array. Only those probes whose expression was detected in at least one of the two conditions (at least 2 out of 3 ES samples/in both the MEF samples) were retained for further analysis. Significance Analysis of Microarrays (SAM)^40^ was applied to the quantile-normalized, log2- transformed expression values. A significance threshold of FDR < 0.001 and absolute log2 fold expression change threshold of 1 were used to identify differentially abundant genes between the two cell types. For every TF, the over-representation of its target set (all genes assigned nonzero TF-gene association scores) in the DEG set was estimated by one-sided Fisher’s exact test. TFs with a p-value of less than 0.05 (after Bonferroni correction for multiple testing) were identified as significantly enriched, and provide hypotheses to explain the transcriptional remodelling that accompanies differentiation. This analysis was carried out separately on the up-regulated and down-regulated genes. As a ‘weighted’ alternative to the above approach that additionally makes use of the TF-gene association scores, we also computed enrichment z-scores for every TF, as follows. First, the sum of its association scores with genes in the DEG set was estimated. Next, subsets of equal size were randomly drawn 1000 times from the full target set of that TF (i.e. all the genes having non-zero association score with it), and the aggregated score of every such sample was recorded. This yielded a baseline distribution of aggregate scores, which was used to compute a z-score (number of standard deviations from the mean) for the DEG set. A large positive z-score was taken to indicate a concordance between the binding site information (regulatory influence of the TF) and expression changes, and thus provides an alternative measure to associate TFs with the genome-wide differential expression.

### Viruses

Lentiviral vectors pLKO.1 NTS Control shRNA was a kind gift from Dr. Manas Santra, pLKO.1 Ezh2 shRNA was a kind gift from Dr. Shravanti Rampalli. These vectors were cotransfected with psPAX2(ADDGENE NO. 12260), pMD2.G (ADDGENE NO. 12259) in HEK 293 T cells using Lipofectamine 2000(Invitrogen, Ref. no. 11668019). Viral supernatants were harvested and used for infection 48 h post transfection.

### *C. elegans* strain

The strain OD95^41^ {unc-119(ed3) III; tIs37 [(pAA64) pie-1p::mCherry::his-58 + unc-119( + )] IV. ltIs38 [pAA1; pie-1::GFP::PH(PLC1delta1) + unc-119( + )]} used to this study were maintained at 20 °C on nematode growth medium (NGM) plates seeded with OP50 *E. coli*.

### RNA interference (RNAi)

*Cav-1* RNAi was carried out by microinjection of *in-vitro* synthesized dsRNAs (Ambion MEGAscript RNAi Kit) into L4 stage hermaphrodites at a concentration ~1 μg/ul using MINJ-1microINJECTORTM (Tritech Research, Inc, CA) and PatchMan^TM^ NP2 micromanipulator (Eppendorf AG,Germany) mounted on a Nikon ECLIPSE Ti-S microscope. Hermaphrodites were dissected 36–48 hours post injection in M9 buffer and appropriate stage embryos were collected, mounted on 3% agarose pads and imaged.

### Microscopy and Image acquisition

*C*. *elegans* embryo imaging was carried out at 20 °C on a Nikon Eclipse Ti-E microscope equipped with a 100x Plan-Apo 1.40 NA objective (Nikon, Tokyo, Japan) and CSU-X1 spinning-disk confocal head (Yokogawa Corporation, Tokyo, Japan). Focus drift during image acquisition was corrected using Nikon’s Perfect Focus System (PFS). Embryo samples were excited using 491 and 568 nm DPSS-Laser (Roper Scientific, France) and images acquired using Prime95B CMOS camera (Photometrics, Tucson, AZ). Image acquisition was controlled by MetaMorph software (Molecular Devices, Sunnyvale, CA). All images are single confocal planes acquired with the camera set at BIN1. Equatorial planes from control or *cav-1 RNAi* embryos co-expressing Histone::mCherry and GFP::PH-PLC1δ were acquired every 10 seconds post anaphase onset. Image analysis was carried out using Fiji.

### Chromatin Immunoprecipitation Assay (ChIP)

mESCs were grown in 10 cm plates at 70% confluency. Cells were fixed for 10 min at 37 °C using a final concentration of 1% formaldehyde in medium. Glycine was added to a final concentration of 125 mM to quench the crosslinking reaction. Cells were washed twice ice cold 1x PBS. 1 ml of cold PBS with protease inhibitors was then added and cells were scraped. 1 ml lysis buffer was added to each plate and incubated at 4 degrees for 3–5 min. Cells were scraped and transferred to 1.5 ml tubes. Chromatin was sheared in BioruptorTM (UCD200) at high setting for a total time of 4 × 5 minutes, 30 seconds ON, 60 seconds OFF. Dynabeads (25ul) were resuspended in 100ul lysate with 900ul of cold dilution buffer, 5 µg of Suz12 antibody or 5 µl of IgG was added to each tube and rotated overnight at 4 °C. Post 4 washes in wash buffer, the immune complexes were eluted by adding 100ul elution buffer to the beads, followed by addition of RNase A and proteinase K at 500 µg/ml and incubated at 37 °C for 1 hour. Crosslinks were reversed at 65 °C overnight. DNA was extracted using phenol-chloroform. RT-qPCR was set up using primers for the *Cav1*, *Cdh2*, *Tgfbr1*, *Tgfbr2* and *Tgfbr3* regions having SUZ12 binding site.

### Data availability

The datasets generated during and/or analysed during the current study are available from the corresponding author on reasonable request.

## Electronic supplementary material

Supplementary Information

## References

[1] Villaseñor R, Kalaidzidis Y, Zerial M (2016). Signal processing by the endosomal system. Curr. Opin. Cell Biol..

[2] Kawamura N (2012). Delivery of endosomes to lysosomes via microautophagy in the visceral endoderm of mouse embryos. Nat. Commun..

[3] Komada M, Soriano P (1999). Hrs, a FYVE finger protein localized to early endosomes, is implicated in vesicular traffic and required for ventral folding morphogenesis. Genes Dev..

[4] Ruland J (2001). p53 Accumulation, defective cell proliferation, and early embryonic lethality in mice lacking tsg101. Proc. Natl. Acad. Sci. USA.

[5] Li L (2010). A unique interplay between Rap1 and E-cadherin in the endocytic pathway regulates self-renewal of human embryonic stem cells. *Stem Cells Dayt*. Ohio.

[6] Song S (2013). Pou5f1-dependent EGF expression controls E-cad endocytosis, cell adhesion, and zebrafish epiboly movements. Dev. Cell.

[7] Sinha A, Khadilkar RJ, S. VK, R. Sinha A, Inamdar MS (2013). Conserved regulation of the Jak/STAT pathway by the endosomal protein asrij maintains stem cell potency. Cell Rep..

[8] Subramanyam D (2011). Multiple targets of miR-302 and miR-372 promote reprogramming of human fibroblasts to induced pluripotent stem cells. Nat. Biotechnol..

[9] Qin H (2014). Systematic identification of barriers to human iPSC generation. Cell.

[10] Boyer LA (2005). Core transcriptional regulatory circuitry in human embryonic stem cells. Cell.

[11] Boyer LA (2006). Polycomb complexes repress developmental regulators in murine embryonic stem cells. Nature.

[12] Lee TI (2006). Control of developmental regulators by Polycomb in human embryonic stem cells. Cell.

[13] Bernstein BE (2006). A bivalent chromatin structure marks key developmental genes in embryonic stem cells. Cell.

[14] Marson A (2008). Connecting microRNA genes to the core transcriptional regulatory circuitry of embryonic stem cells. Cell.

[15] Wang Y (2008). Embryonic stem cell-specific microRNAs regulate the G1-S transition and promote rapid proliferation. Nat. Genet..

[16] Chen X (2008). Integration of external signaling pathways with the core transcriptional network in embryonic stem cells. Cell.

[17] Mikkelsen TS (2008). Dissecting direct reprogramming through integrative genomic analysis. Nature.

[18] Mas G, Di Croce L (2016). The role of Polycomb in stem cell genome architecture. Curr. Opin. Cell Biol..

[19] Laugesen A, Helin K (2014). Chromatin repressive complexes in stem cells, development, and cancer. Cell Stem Cell.

[20] Judson RL, Babiarz JE, Venere M, Blelloch R (2009). Embryonic stem cell-specific microRNAs promote induced pluripotency. Nat. Biotechnol..

[21] Bartel DP (2009). MicroRNAs: target recognition and regulatory functions. Cell.

[22] Wang Y, Medvid R, Melton C, Jaenisch R, Blelloch R (2007). DGCR8 is essential for microRNA biogenesis and silencing of embryonic stem cell self-renewal. Nat. Genet..

[23] Cheng JPX, Nichols BJ (2016). Caveolae: One Function or Many?. Trends Cell Biol..

[24] Di Guglielmo GM, Le Roy C, Goodfellow AF, Wrana JL (2003). Distinct endocytic pathways regulate TGF-beta receptor signalling and turnover. Nat. Cell Biol..

[25] Sotgia F (2005). Caveolin-1-Deficient Mice Have An Increased Mammary Stem Cell Population with Upregulation of Wnt/?-Catenin Signaling. Cell Cycle.

[26] Parton RG, del Pozo MA (2013). Caveolae as plasma membrane sensors, protectors and organizers. Nat. Rev. Mol. Cell Biol..

[27] Kawauchi T (2010). Rab GTPases-dependent endocytic pathways regulate neuronal migration and maturation through N-cadherin trafficking. Neuron.

[28] Frampton J, Fukuda K, Teramura T, Takehara T, Onodera Y (2015). Cdh2 stabilizes FGFR1 and contributes to primed-state pluripotency in mouse epiblast stem cells. Sci. Rep..

[29] Haque A, Yue X-S, Motazedian A, Tagawa Y, Akaike T (2012). Characterization and neural differentiation of mouse embryonic and induced pluripotent stem cells on cadherin-based substrata. Biomaterials.

[30] Haque A (2015). An Engineered N-Cadherin Substrate for Differentiation, Survival, and Selection of Pluripotent Stem Cell-Derived Neural Progenitors. PLOS ONE.

[31] Scheel J, Srinivasan J, Honnert U, Henske A, Kurzchalia TV (1999). Involvement of caveolin-1 in meiotic cell-cycle progression in Caenorhabditis elegans. Nat. Cell Biol..

[32] Boosani CS, Agrawal DK (2015). Methylation and microRNA-mediated epigenetic regulation of SOCS3. Mol. Biol. Rep..

[33] Zhang H, Emmons SW (2009). Regulation of the *C. elegans* posterior Hox gene egl-5 by microRNA and the Polycomb-like gene sop-2. Dev. Dyn. Off. Publ. Am. Assoc. Anat..

[34] Chen Y-T, Tai C-Y (2017). μ2-Dependent endocytosis of N-cadherin is regulated by β-catenin to facilitate neurite outgrowth. Traffic Cph. Den..

[35] Itoh F, Watabe T, Miyazono K (2014). Roles of TGF-β family signals in the fate determination of pluripotent stem cells. Semin. Cell Dev. Biol..

[36] Li Z, Yang C-S, Nakashima K, Rana TM (2011). Small RNA-mediated regulation of iPS cell generation. EMBO J..

[37] Chen Y-G (2009). Endocytic regulation of TGF-β signaling. Cell Res..

[38] Hayer A (2010). Caveolin-1 is ubiquitinated and targeted to intralumenal vesicles in endolysosomes for degradation. J. Cell Biol..

[39] Kittler R (2007). Genome-wide resources of endoribonuclease-prepared short interfering RNAs for specific loss-of-function studies. Nat. Methods.

[40] Tusher VG, Tibshirani R, Chu G (2001). Significance analysis of microarrays applied to the ionizing radiation response. Proc. Natl. Acad. Sci. USA.

[41] Audhya A (2005). A complex containing the Sm protein CAR-1 and the RNA helicase CGH-1 is required for embryonic cytokinesis in Caenorhabditis elegans. J. Cell Biol..

