# Supplementary material for: Dual repression of endocytic players by ESCC microRNAs and the Polycomb complex regulates mouse embryonic stem cell pluripotency
Source: Sci Rep. 2017 Dec 14;7:17572. doi: 10.1038/s41598-017-17828-7 (PMC5730570; doi:10.1038/s41598-017-17828-7)
Supplement: Supplementary file 1 — Supplementary Information [file 41598_2017_17828_MOESM1_ESM.pdf]

Dual repression of endocytic players by ESCC microRNAs and the Polycomb complex regulates mouse embryonic stem cell pluripotency

Ridim Dadasaheb Mote, Gaurang Mahajan, Anup Padmanabhan, Ramaraju Ambati and Deepa Subramanyam*

Supplemental Figure 1

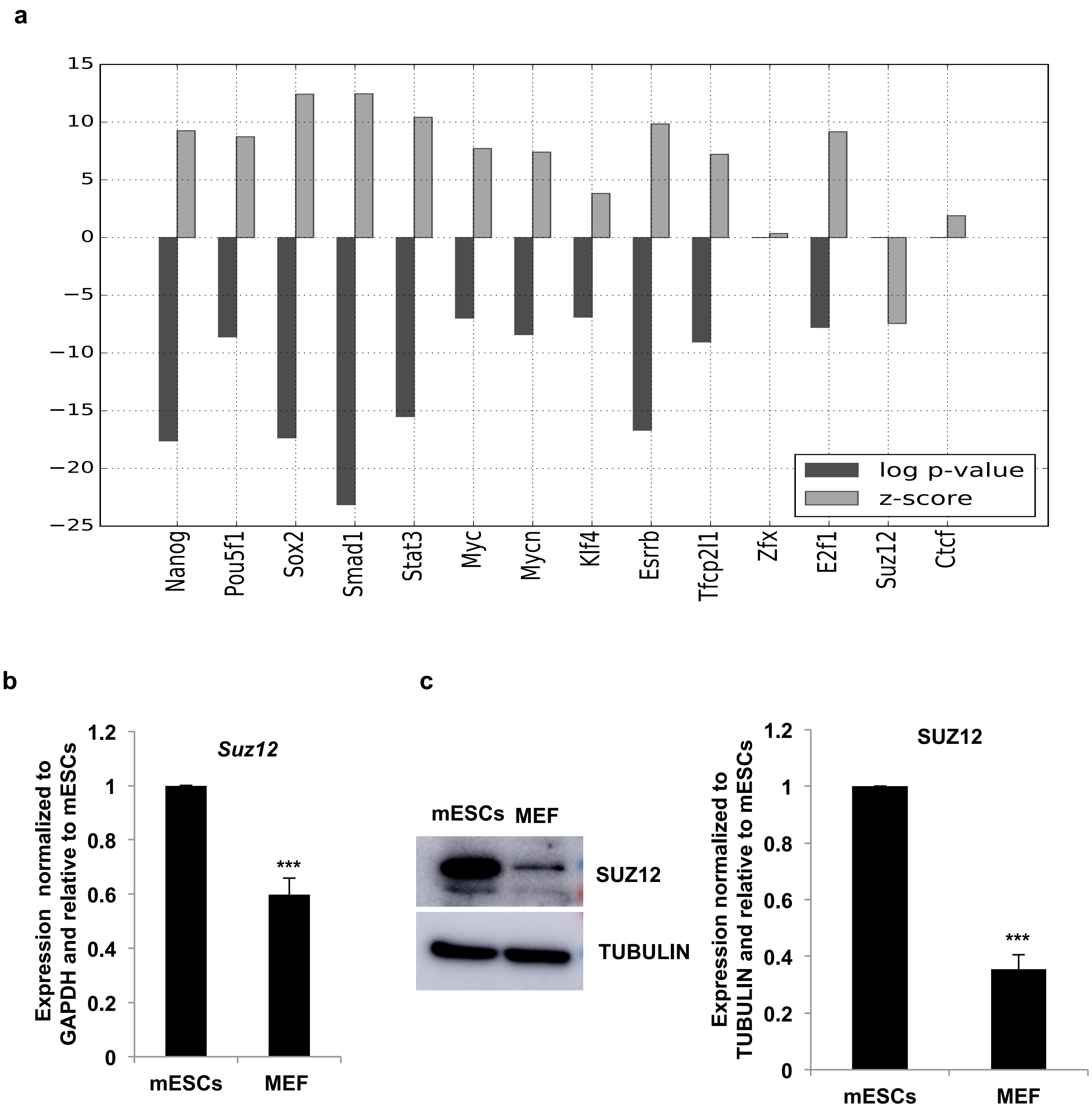

Supplemental Figure 1: Pluripotency network mediated regulation in mESCs. a) Bar chart displaying the statistical association between gene sets bound by different transcriptional regulators (from ChIP-seq binding data in Chen et al.) and the set of genes whose expression levels are >2 fold higher in mESCs relative to MEFs. Differentially up-regulated genes are significantly over-represented among the target sets of 11 out of the 13 sequence-specific transcription factors (indicated by small p-values and high Z-scores). b**)** RT-qPCR analysis showing levels of *Suz12* in mESCs and MEFs. **c)** Western blot showing expression of SUZ12 in mESCs and MEFs (N=3). Quantitation of the Western blot is shown to the right. Error bars represent mean ± S.D (N=3). * * * p< 0.001 by Students T-test.

Supplemental Figure 2

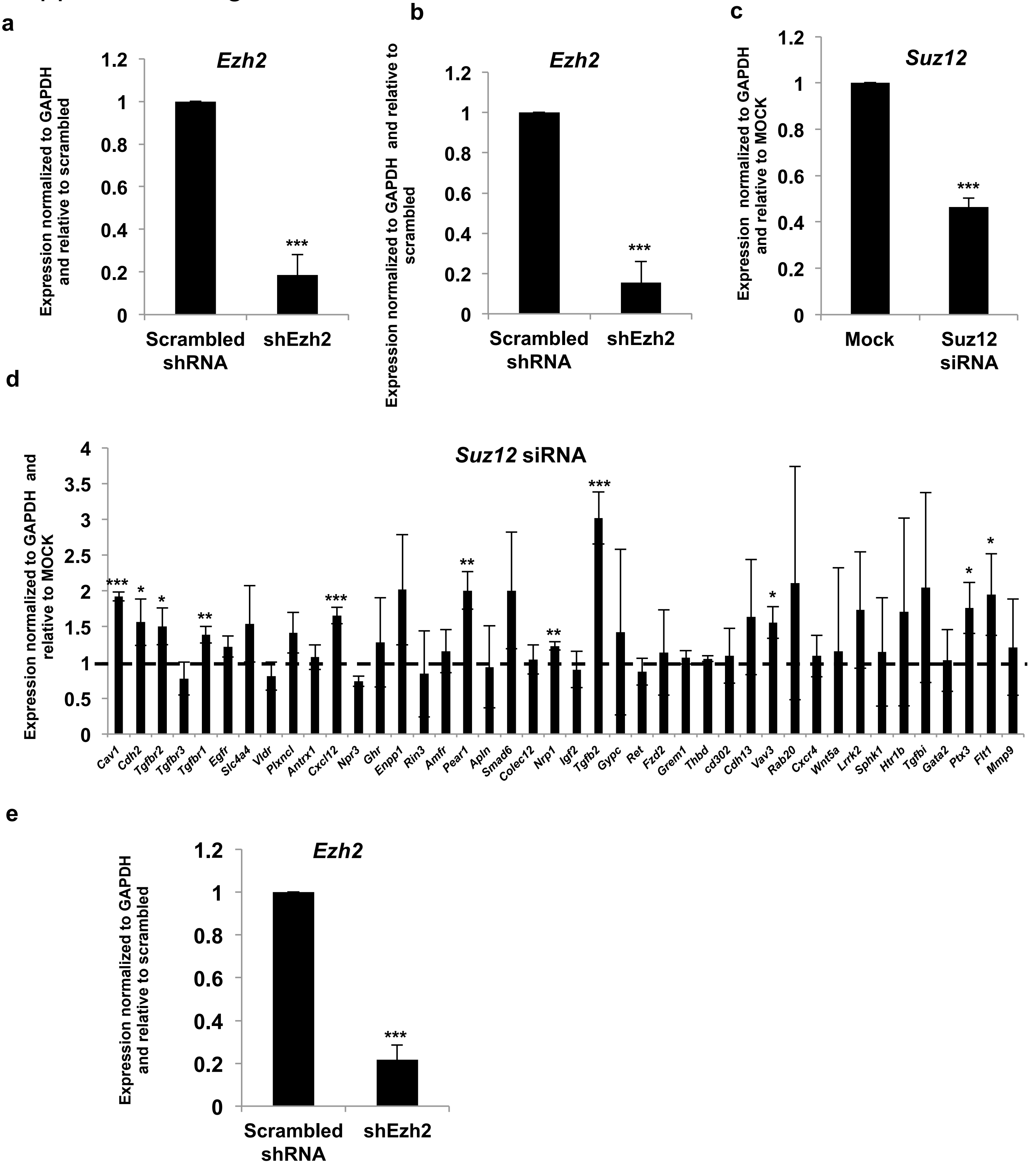

Supplemental Figure 2: Knockdown of PRC2 complex components leads to derepression of endocytic genes in mESCs. a) & b) RT-qPCR analysis showing knockdown of *Ezh2* mRNA levels in V6.5 and R1 mESCs respectively, upon sh*Ezh2* lentivirus transfection (N=3). * * * p < 0.001 by Student's T-test. **c)** RT-qPCR analysis showing knockdown of *Suz12* mRNA levels in mESCs 48 hours post-transfection with *Suz12* siRNA (N=3), * * * p < 0.001 by Student's T-test. **d)** RT-qPCR analysis of PRC2 complex target genes upon *Suz12* knockdown in mESCs. Error bars represent mean ± S.D for experiments in triplicates (N=3). * p< 0.05; * * p < 0.01; * * * p < 0.001 by Students T-test. mRNA expression is normalized to *Gapdh* and represented as relative to mock. **e)** RT-qPCR analysis showing levels of *Ezh2* mRNA in *Dgcr8* KO mESCs upon sh*Ezh2* lentivirus transfection (N=3), * * * p < 0.001 by Student's T-test.

Supplemental Figure 3

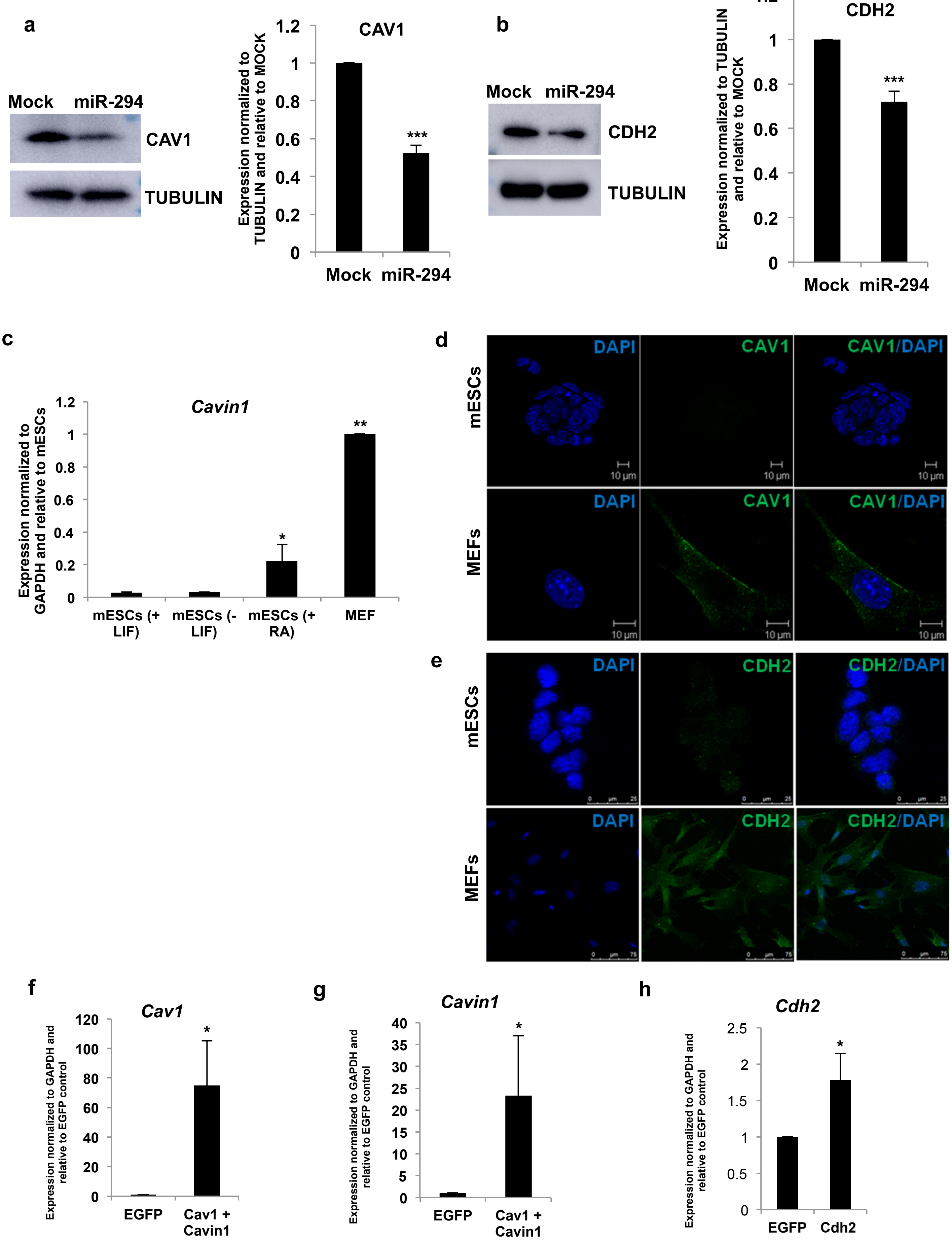

Supplemental Figure 3: Regulation of Cav1, Cdh2 expression by ESCC miRNAs. a, b) Western blot showing levels of CAV1 (a), and CDH2 protein (b) in HEK293 cells in the presence or absence of exogenous miR-294, 48 hrs post-transfection. Quantitation of the Western blot is shown to the right of each blot. Error bars represent mean ± S.D (N=3). * * * p< 0.001 by Students T-test. **c)** RT-qPCR analysis for *Cavin1* mRNA expression in the following samples: mESCs cultured in the presence of LIF, mESCs cultured in the absence of LIF for 72hrs, mESCs cultured with retinoic acid for 96hrs, and in MEFs (N=3). **d)** Representative micrographs showing immunofluorescence staining for CAV1 in mESCs (top), and in MEFs (bottom). The scale bar represents 10 µm. **e**) Representative micrographs showing immunofluorescence staining for CDH2 in mESCs (top), and in MEFs (bottom). The scale bar represents 10 µm. **f, g & h)** RT-qPCR analysis showing levels of *Cav1*, *Cavin1* and *Cdh2* after overexpression in mESCs (N=3). * p< 0.05 by Students T-test.

Supplemental Table 1: Table showing the presence of seed matches to miR-294 in targets of SUZ12

Sr. no.	Genes	8mer site	7mer-m8 site	7mer- A1 site	6mer site
1	*Nrp1*	-	-	-	-
2	*Antxr1*	-	-	-	-
3	*Cav1*	-	-	-	1 (3'UTR)
4	*Gdnf*	-	1 (3'UTR)	-	-
5	*Igf2*	-	-	-	-
6	*Tac1*	-	-	-	-
7	*Egfr*	-	-	-	1(ORF) 2(3'UTR)
8	*Cxcl12*	-	-	-	-
9	*Tgfb2*	-	-	-	-
10	*Npr3*	-	1 (3'UTR)	-	-
11	*Tgfbr2*	1 (3'UTR)	1 (3'UTR)	-	1 (3'UTR)
12	*Gypc*	-	-	-	-
13	*Adra2a*	-	1 (3'UTR)	-	-
14	*Ghr*	-	-	-	-
15	*Slc4a4*	-	-	1 (ORF)	-
16	*Enpp1*	-	-	-	1 (3'UTR)
17	*Rin3*	-	-	-	-
18	*Tgfbr3*	-	-	-	1 (ORF)
19	*Ret*	-	-	-	-
20	*Fzd2*	-	-	-	-
21	*Grem1*	-	-	-	1 (3'UTR)
22	*Vldlr*	-	1 (3'UTR)	-	2 (3'UTR)
23	*Amfr*	-	-	-	-
24	*Pear1*	-	1 (ORF)	-	1 (3'UTR)
25	*Sim2*	-	-	-	-
26	*Thbd*	-	-	-	-
27	*Cd302*	-	-	-	-
28	*Cdh13*	-	-	-	-
29	*Plxnc1*	1 (3'UTR)	-	-	2 (ORF)
30	*Vav3*	-	-	-	-
31	*Apln*	-	-	-	-
32	*Rab20*	-	-	-	-
33	*Smad6*	-	-	-	-
34	*Cxcr4*	-	-	1 (3'UTR)	-
35	*Slc18a2*	-	-	-	2 (3'UTR)
36	*Wnt5a*	-	-	-	-
37	*Cd34*	-	-	-	-
38	*Lrrk2*	-	-	-	2 (ORF)
39	*Sphk1*	-	-	-	-
40	*Ntf3*	-	-	-	-
41	*Tgfbr1*	-	1 (3'UTR)	1 (ORF)	1 (ORF)
42	*Htr1b*	-	-	-	-
43	*Tgfbi*	-	-	-	-
44	*Gata2*	-	-	-	-
45	*Cdh2*	-	1 (ORF)	-	-
46	*Ptx3*	-	-	-	-
47	*Flt1*	-	1 (3'UTR)	-	-
48	*Colec12*	-	-	-	-
49	*Mmp9*	-	-	-	1 (3'UTR)
50	*Dnm3*	-	-	-	1 (3'UTR)

Supplemental Table 2: List of primers used in this study

Sr. no.	Gene	Forward primer	Reverse primer
1	*Vldlr*	ACTGAGACAATGGCGTGATATAG	AACACACACCGACAGTCATAG
2	*Vav3*	GAAGTTTACTCTGCGCGATTTG	CATAGGGTCATGGGTGTGTTT
3	*Thbd*	GAGCCAGAGCAGTATGGATTTAG	TGCTGTAACTGGTGTGGTTATC
4	*Tgfbr3*	CTCCAACCATCAGCCTTTCT	ACTCCCTGTCTCTGTATCATCT
5	*Tgfbr2*	GTTCGTGAGCATGGAGAGATAG	CAGGGCTGAGATGATAAGAGTG
6	*Tgfb2*	GGCTTTCATTTGGCTTGAGATG	CTTCGGGTGAGACCACAAATAG
7	*Tac1*	CATGGCCAGATCTCTCACAAA	GCATCGCGCTTCTTTCATAAG
8	*Smad6*	CCTAGCAGCTCTTTGGGATTT	AAGCAATTCCGTCTCAGGTC
9	*Slc4a4*	GGTATGTGGCTGCTACTGTTAT	GTGACTCGTTGTTCCCTTACTC
10	*Slc18a2*	CCGTCGGTGATGATGAAGAA	CAATGGATGGCGTGACTAAGA
11	*Sim2*	AGCTCACAGGCAACAGTATTTA	AGACCGCTCTATCTCGTACTC
12	*Rin2*	CTCTGAAGACAAGGATGGGAAG	CAGCCATATGGGATGGGTATG
13	*Ret*	CAATGAGACTACTGGCCTTCTC	CAGAAAGACCTGGAGGAAGATG
14	*Rab20*	GTACAAGATGCTGGATGAGAGG	GGTACCACCAGGTCAAACAA
15	*Plxncl*	ACTGGATGTCTGTCTGTCTCT	CCACGGGACCCTTGTTTATT
16	*Pear1*	GAGGGAGTCAGGTATAGGTTCT	CATGGAGGATTACTGGGTGAAG
17	*Nrp1*	GAGGACAGAGACTGCAAGTATG	CTGAAGACACCACAGGAGAAG
18	*Npr3*	CAGTGGAGACTACGCTTTCTT	AGGGACGAGTATGCTTGTTTAG
19	*Igf2*	ACTGAGACAATGGCGTGATATAG	AACACACACCGACAGTCATAG
20	*Gypc*	GAAGTTTACTCTGCGCGATTTG	CATAGGGTCATGGGTGTGTTT
21	*Grem1*	GAGCCAGAGCAGTATGGATTTAG	TGCTGTAACTGGTGTGGTTATC
22	*Ghr*	CTCCAACCATCAGCCTTTCT	ACTCCCTGTCTCTGTATCATCT
23	*Gdnf*	GTTCGTGAGCATGGAGAGATAG	CAGGGCTGAGATGATAAGAGTG
24	*Fzd2*	GGCTTTCATTTGGCTTGAGATG	CTTCGGGTGAGACCACAAATAG
25	*Enpp1*	CCAGTCCCAGTTCCAAAGATTA	TTGTAGAAGTCGGGTTTGTAGTT
26	*Egfr*	GGACAAAGGCAGAGACGATAAA	CACACTCTGTGTCCAGAATCAA
27	*Cxcr4*	CGACGGCTCTATGTTCTTCTC	TCGCTGCATGTCCACTAAATA
28	*Cxcl12*	GGTGGACTTCTTCCTGTCATTAG	GCCATGGGATTCCGGATAAA
29	*Cdh13*	GGAGAGAATCCCTTTGGAGAAC	CCAGTTCTGTTTGTCCCATAGT
30	*Cd302*	GAAGTGGGTTCTGGAGACTATG	CGACTATGCCAGTCAAGAAGAT
31	*Cav1*	GTTGCTTGGGAGGAAACAAATAC	CACAAATCCGGCTCCCTAAA
32	*Apln*	GGAGTGGATCAAGACCCTAAAG	TCCGTGGTTTCCACATACAG
33	*Antxr1*	CTCCTGCTAGGCATGTTCTATG	CAACTAGGTCCTCACCATCTTG
34	*Amfr*	GTGACTGACTGGGATGATTTAGG	GCTTGGCAGTCTCGGTTTA
35	*Adra2a*	CACCCACCAAGTTCCTCTAATG	CATGGAACAGGTCAAGGAGAAG
36	*Wnt5a*	CAGGCTCTCCAAGGCATTAT	TCGGAAGCCATTTCCTCTTAC
37	*Tgfbr1*	CCTTGAGTCACTGGGTGTTATG	CCACTTAGCTGTCACCCTAATC
38	*Tgfbi*	GACCACAAGAACGAGGAGATG	TCTCCAGTAACCGCTGATAGA
39	*Sphk1*	GGTACGAGCAGGTGACTAATG	GGACAGACTGAGCACAGAATAG
40	*Ntf3*	CCTGGAAATAGTCACACGGATG	CTTGGATGCCACGGAGATAAG
41	*Lrrk2*	AGAGCCTGCGATCAGAATAAC	GGACTGCTCTCTTTCTCACATAC
42	*Htr1b*	CCTGGTGATGCCTATCTGTAAG	CCTCATTGGACATGGTGTAGAT
43	*Gata2*	ATACCCACCTATCCCTCCTATG	AGCCTTGCTTCTCTGCTTAG
44	*Cd34*	CAGACCCTTCTGTGCCTTATC	CATGTTGATGCGCTGGATTG
45	*Ptx3*	AGGGTGGACTCCTACAGATT	TGAGAACCCGATCCCAGATA
46	*Mmp9*	CAATCCTTGCAATGTGGATGTT	CCTGTAATGGGCTTCCTCTATG
47	*Flt1*	ACATGGGAAGACAGGGTAGA	GACCTAAGCACACAGACCATAA
48	*Dnm3*	GAAGAGTGTGGACCTGGTAATG	GTCCTTTGTCTTCCCTTCTCTC
49	*Colec12*	AGGACACCGATTGGCTAAAG	ATGAGCTGAGCTGGCTATTC
50	*Cdh2*	GGATGAAACGGCGGGATAAA	TCTTCTTCTCCTCCACCTTCTT
51	*Cavin1*	CCAAGGAGAAGATGGAGAAGAC	CAGCTTGTTCATGCGCTTC
52	*Cav1* 3'UTR	GCGTACTCGAGGGGACATTTCAAGGATG	TAATAGCGGCCGCCCTCCCTGGAGGTTC
53	*Cdh2 ORF*	GCGTACTCGAGATGGAATCCCGCCTATGAGT	CAGATGCGGCCGCCTTCATAGATACCAGCTTCC
54	*Gata6*	AGCAAGATGAATGGCCTCAG	CTCACCCTCAGCATTTCTACG
55	*Suz12*	TGGGAGACAATTCTTGATGGG	GGAGCTGTAGACTTATCGTTGG
56	*Snail*	GCTGATGGAGTGCCTTTGTA	CCAGTGGGTTGGCTTTAGTT

Sr. no.	Gene	Forward primer	Reverse primer
57	*Keratin19*	CTCCCGAGATTACAACCACTAC	GTTCTGTCTCAAACTTGGTTCTG
58	*Oct4*	AAAGCCCTGCAGAAGGAGCTAGAA	AACACCTTTCCAAAGAGAACG
59	*Sox2*	ACTTTTGTCCGAGACCGAGAA	CGCGGCCGGTATTTATAATC
60	*Nanog*	GCTCAGCACCAGTGGAGTATCC	TCCAGATGCGTTCACCAGATAG
61	*Klf4*	GACCTCCTGGACCTAGACTTTA	GAAGACGAGGATGAAGCTGAC
62	*Gapdh*	AACAGCAACTCCCACTCTTC	CCTGTTGCTGTAGCCGTATT
63	*Sox1*	CTTCATGGTGTGGTCCCG	TTGCTGATCTCCGAGTTGTG
64	*BetaIII tubulin*	CAGGCCCGACAACTTTATCT	CTCTTTCCGCACGACATCTA
65	*Nestin*	AAGTTCCCAGGCTTCTCTTG	GTCTCAAGGGTATTAGGCAAGG
66	*Ezh2*	CGCGGGACGAAGAATAATCA	GCTGTCTCAGTCTCATGTACTC
67	*Human cav1*	TGCATCAGCCGTGTCTATTC	GCAAGTTGATGCGGACATTG
68	*Suz12_siRNA*	GGGCGGGTGATGGCTCCTATGCAGGAAA	GGGCGGGTCAAACAGCATACAGGCATGA
69	*Gfp_siRNA*	GGGCGGGTATGGTGAGCAAGGGCGAGGAG	GGGCGGGTTTACTTGTACAGCTCGTCCATG
70	*T7*	TAATACGACTCACTATAGGGAGACCACGGGCGGGT	TAATACGACTCACTATAGGGAGACCACGGGCGGGT
